# Compressive Mechanical Properties of Lattice Structures with Varied Structural Parameters Prepared by Stereolithography

**DOI:** 10.3390/ma18163898

**Published:** 2025-08-20

**Authors:** Jianhua Sun, Hai Gu, Jie Zhang, Guoqing Dai, Bin Li, Zhonggang Sun, Zulei Liang

**Affiliations:** 1 School of Mechanical Engineering, Nantong Institute of Technology, Nantong 226001, China; sjhuant@ntit.edu.cn (J.S.); zhangjie@ntit.edu.cn (J.Z.); libin19@ntit.edu.cn (B.L.); zuleiliang@163.com (Z.L.); 2 Jiangsu Key Laboratory of 3D Printing Equipment and Application Technology, Nantong Institute of Technology, Nantong 226001, China; guoqingdai@njtech.edu.cn (G.D.); sunzgg@njtech.edu.cn (Z.S.); 3 College of Materials Science and Technology, Tech Institute for Advanced Materials, Nanjing Tech University, Nanjing 210009, China

**Keywords:** lattice structure, structural parameters, mechanical properties, stereolithography

## Abstract

The use of additive manufacturing technology for the lightweight design of complex lattice structures is becoming increasingly popular, but research on lattice structure design and strength evaluation still relies on the visual comparison of stress distributions and lacks quantitative assessment data. Given this perspective, this study explored the effects of structural parameters (relative density, cell size, and sample size) on the compressive strength of diamond lattice structures prepared by Stereolithography (SLA) and revealed the underlying mechanisms through stress distribution simulations and the calculation of characteristic stress distribution parameters (structural efficiency and stress concentration coefficient). The results showed that a greater relative density can increase structural efficiency, but it hardly affects the stress concentration coefficient, and smaller cell sizes and larger sample sizes increase the stress concentration coefficient without affecting the structural efficiency. Lattice structures with a greater relative density, higher structural efficiency, and a larger stress concentration coefficient exhibit higher compressive strength according to the lattice strength formula, which indicates that lattice strength is determined by the product of structural efficiency, stress concentration coefficient, relative density, and material strength. The relevant conclusions could guide the analysis of lattice stress distribution and the design of lattice structures.

## 1. Introduction

The demand for lightweight structures with high specific strength [1], high energy absorption [2], controllable elastic modulus [3], and multifunctional capabilities has spurred advancements in lattice structures, which are crucial in aerospace, automotive, and biomedical engineering [4,5]. Lattice structures or lattice metamaterials have thus garnered a lot of attention due to their excellent mechanical properties and high design flexibility [6,7]. Compared with the bulk structure, the lattice structure reduces the self-weight of the parts without decreasing the mechanical properties and simultaneously provides additional space for multifunctional integration [8,9]. Traditional manufacturing methods, such as powder metallurgy and precision casting, are unable to produce lattice structures with complex geometries and frequently lead to issues like closed pores and crack defects. Additive manufacturing (AM) has transformed prototyping and production by enabling the creation of complex geometries unattainable through conventional methods [10]. Among AM technologies, Stereolithography (SLA) is particularly well-suited for rapid prototyping owing to its compact size, low cost, and minimal environmental requirements. Therefore, SLA enables the precise fabrication of lattice structures, ensures the consistency of their mechanical properties, and supports in-depth research into lattice structures [11,12].

According to previous research [13,14,15,16,17], lattice structures exhibit mechanical properties that can differ significantly from those of the bulk material, primarily depending on their spatial configurations and structural parameters. Leary et al. [18] investigated the effects of different cell types and cell sizes on the deformation behavior of Ti6Al4V lattice structures under compressive loading and found that the failure mode of the lattice structure subjected to compressive loading is the buckling of struts, which is subsequently followed by strut fracture and the collapse of the entire lattice along the diagonal shear zone. Li et al. [19] and Liu et al. [20] found that the body-centered cubic (BCC) lattice structure has a lower elastic modulus and specific strength than the face-centered cubic (FCC) lattice structure, and an increase in cell size results in reductions in specific modulus and specific strength. Wei et al. [21] and Ahmadi et al. [8] systematically studied the deformation characteristics of various lattice structures and found that the spatial configuration of the lattice structure largely determines the degree of local stress concentration.

Structural improvement is a common method used to improve the mechanical properties of lattice structures. Zhao et al. [22,23] optimized the lattice nodes with arc transitions using the triply periodic minimal surface (TPMS) design method and proposed a parametric modeling method for conical struts, which alleviated the local stress concentration of the lattice structure to a certain extent. Bai et al. [24] proposed an improved lattice structure scheme that changed linear struts to arc-shaped struts, which also alleviated the local stress concentration at the nodes. Zhao et al. [25] theoretically calculated and optimized the service orientation of the struts of the body-centered cubic (BCC) lattice and found that under the uniaxial compression service condition, the optimal service orientation of the BCC lattice struts was approximately 71.76°. In addition, since the vertical struts with an angle of 0° to the external load theoretically only bear axial forces, their stress distribution in the length direction is relatively uniform, which can effectively share the service stress in the node area. Thus, improved lattice structures such as BCCZ and FCCZ were developed [26,27,28].

It can be assumed that spatial configurations and structural parameters determine the mechanical properties of lattice structures. However, current studies have primarily focused on specific spatial configurations [29,30,31,32], such as the simple cubic (SC) lattice, BCC lattice, and FCC lattice, as well as various loading conditions [33,34,35,36]. The exploration of structural parameters and their resultant mechanical properties has been relatively limited, leading to the underlying influence mechanisms of structural parameters (relative density, cell size, and sample size) on their mechanical properties remaining insufficiently understood. Therefore, a comprehensive and detailed investigation is necessary to explore the effects and underlying influence mechanisms of structural parameters on the mechanical properties of lattice structures.

In this study, a diamond structure was selected as the research object, and the SLA process was used to prepare the lattice samples. Then, the compressive mechanical properties of the lattice samples were investigated through experiments, the stress distributions were simulated using the finite element method (FEM), and the influence mechanism of these structural parameters was clarified by calculating the variation trend of the structural efficiency and stress concentration coefficient.

## 2. Materials and Methods

### 2.1. Sample Preparation

**Figure 1:**
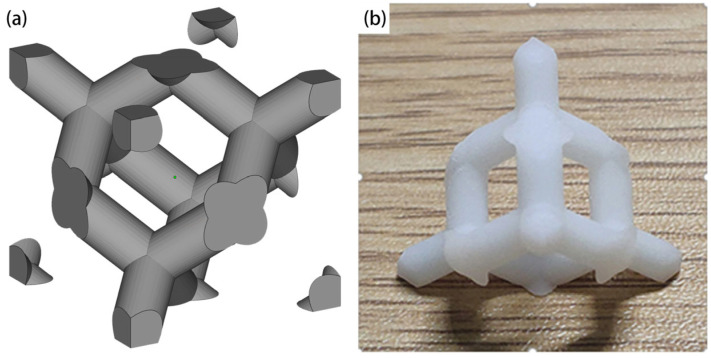
The diamond lattice structure. (**a**) CAD model of the diamond lattice cell. (**b**) A diamond lattice sample fabricated by the SLA process.

A diamond structure was selected due to its uniform structure and excellent mechanical properties, as shown in Figure 1, where the inclined angle of all struts is 35.3°. The computer-aided design (CAD) models of lattice structures were designed in SolidWorks 2023 and converted to STL format. All samples were printed using KS-3860 hard UV resin (Kongshi, Dongguan, China). The SLA process involves the use of a laser with a specific wavelength and a specific power, which is focused on the surface of the light-curing material, enabling the sequential solidification of photosensitive resin. This process commences with the creation of lines, wherein multiple adjacent lines overlap to form a surface. Subsequently, the workbench descends vertically by one layer thickness, allowing for the preparation of a new layer. In this manner, the layers are accumulated to fabricate three-dimensional structures, such as lattice structures.

**Figure 2:**
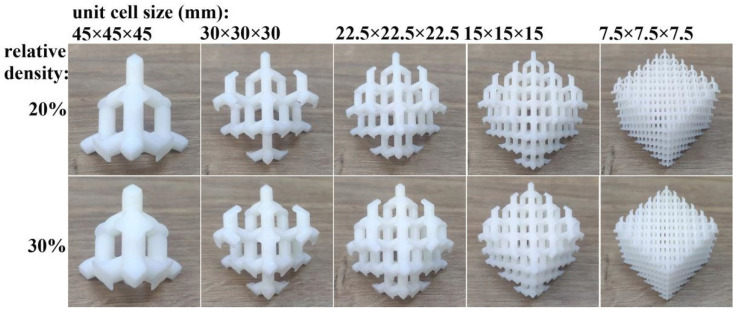
Diamond lattice samples with different cell sizes and different relative densities (sample size is 45 mm).

**Figure 3:**
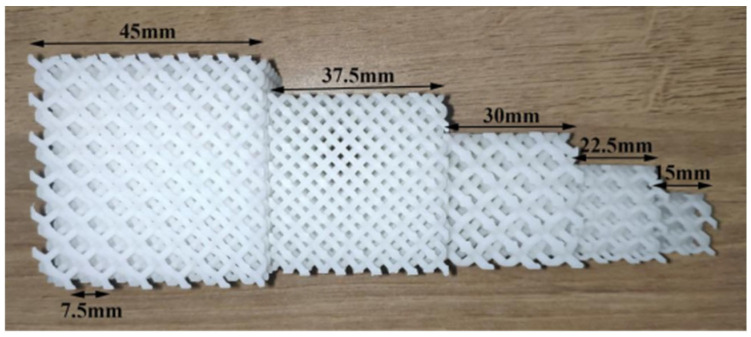
Diamond lattice samples with different sample sizes (cell size is 7.5 mm, and relative density is 20%).

In this work, 3DSL-360S (Creality, Ningbo, China) was used as the SLA additive manufacturing machine; the layer thickness was set to 0.1 mm, the scanning speed was 8 mm/s, the hatch spacing was 0.10 mm, and the laser compensation was 0.18 mm. Lattice structures with different relative densities, cell sizes, and sample sizes were designed and printed by the SLA machine. The structuring parameters are listed in Table 1, and the lattice samples are shown in Figure 2 and Figure 3. All the lattice samples were printed and tested under compression following the placement method shown in Figure 2 and Figure 3. After the lattice samples were printed, they were cleaned with industrial alcohol to remove the uncured resin on the surface.

### 2.2. Mechanical Property Testing

**Figure 4:**
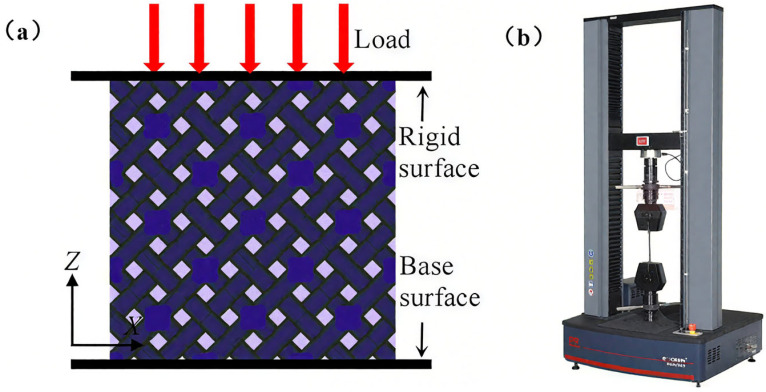
Schematic diagram of compression test (**a**) and the testing machine (**b**).

Figure 4a shows a schematic diagram of the compressive test of a lattice structure. Compression tests were performed using an E43.504 electronic universal testing machine (MTS, Eden Prairie, MN, USA) with a loading rate of 1.0 mm/min (Figure 4b). The machine is capable of measuring forces up to 50 kN with a precision of ±0.1%, while displacement was recorded with a resolution of 0.01 mm. Tests were conducted at 25 °C and lasted approximately 10 min. The testing machine recorded real-time force and displacement data, which were subsequently utilized to construct stress–strain curves. The compression test followed the ASTM D695 standard [37]. Each group of samples was tested three times in the building direction.

### 2.3. Finite Element Modeling

The finite element (FE) simulations were performed using the commercial software ABAQUS/Explicit 6.13. The lattice structure was placed between rigid upper and lower platens. All degrees of freedom were restricted for the lower platen, while the upper platen was displaced downward at a predefined velocity, with its other degrees of freedom also constrained. Taking into account the inertia effects and computational costs, the loading speed of the platen in the simulations was established at 1 m/s. A penalty contact algorithm (built-in in the ABAQUS/Explicit 6.13) with a friction coefficient of 0.2 was used between the lattice structure and the platens, as well as within the lattice structure itself. The lattice structure was meshed using C3D10M elements, whereas R3D4 elements were employed for meshing the platens. The element size for the lattice structure was determined to be 1/4 of the strut diameter, and for the platens, it was set to 1 mm. Physical properties of the photosensitive resin used in Abaqus models are listed in Table 2.

### 2.4. Formulation of Structural Efficiency and Stress Concentration Coefficient

The Von Mises stress of lattice materials is approximately periodically distributed due to the periodic arrangement of lattice cells. When the volume element *dV* is near the upper surface, its displacement strain $\varepsilon_{d}$ is close to the apparent strain $\varepsilon_{a}$. For the volume element *dV* far from the upper surface, the displacement strain $\varepsilon_{d}$ approaches zero. Therefore, it can be considered that within a specific range of Von Mises stress, the corresponding displacement strain $\varepsilon_{d}$ is approximately proportional to the apparent strain $\varepsilon_{a}$ and is independent of the Von Mises stress value.

(1) $$\varepsilon_{a}\approx \;C_{L}\varepsilon_{d}$$

Here, *C_L_* is a constant related to the spatial structure of the lattice.

To further quantitatively analyze the influence of the lattice stress distribution on the mechanical properties of the lattice, the lattice structure efficiency and the stress concentration coefficient were calculated using a method outlined in our previous study [38]. The formula of the structural efficiency $\frac{1}{C_{L}}$ can be written as follows:

(2) $$\frac{1}{C_{L}}=\frac{\sigma_{a}}{∭\sigma d\frac{V}{Al}}$$

Here, $\sigma_{a}$ is the apparent stress of the lattice, σ is the Von Mises stress of a certain volume element in the lattice, *V* is the volume of the lattice, *A* is the force-bearing area of the lattice, *l* is the height of the lattice, and *Al* is the apparent volume of the lattice. $∭\sigma d\frac{V}{Al}$ can be calculated by the Von Mises–volume curve, which can be extracted from the Abaqus Odb files using a Python 2.6 script (https://github.com/Zuleiliang/Mises-Volume-curve-for-lattice.git, accessed on 1 June 2025).

The formula of the stress concentration coefficient $K_{t}$ can be written as follows:

(3) $$K_{t}=\frac{Al}{V\sigma_{max}}∭\sigma d\frac{V}{Al}$$

Here, $\sigma_{max}$ is the maximum Von Mises stress in the lattice. When the lattice is about to fracture, $\sigma_{max}$ can also be considered indicative of the material strength.

According to Formulas (2) and (3), the formula for the apparent stress $\sigma_{a}$ of the lattice is as follows:

(4) $$\sigma_{a}=\frac{1}{C_{L}}\times K_{t}\times \frac{V}{Al}\times \sigma_{max}$$

When the value of the apparent stress $\sigma_{a}$ reaches the maximum, the apparent stress is the lattice strength. V/Al is the relative density, and $\sigma_{max}$ can be considered the material strength. Therefore, the lattice strength is the product of the structural efficiency, the stress concentration coefficient, the relative density, and the material strength [38].

According to Formula (2), the structural efficiency is the ratio of the lattice apparent stress to the integral of material stress over lattice volume. When the integral of material stress over lattice volume is the same, a larger lattice apparent stress results in higher structural efficiency. According to Formula (3), the stress concentration coefficient is the ratio of the maximum stress of the lattice to the average stress of the lattice. The average stress is obtained by dividing the integral of the material stress over the lattice volume by the lattice volume.

## 3. Results and Discussion

### 3.1. Mechanical Properties of Diamond Lattice Structures

**Figure 5:**
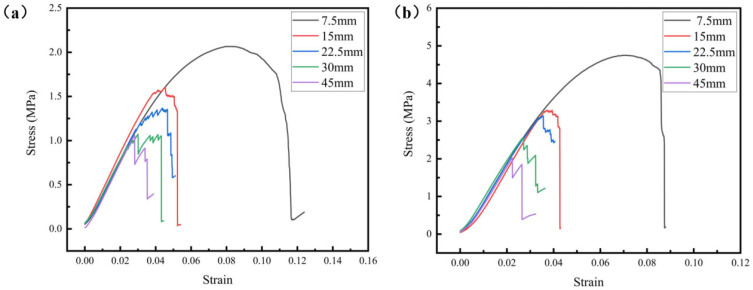
The compression curves of diamond lattices with a relative density of 20% (**a**) and 30% (**b**).

The cell type of the lattice structure has a direct impact on its mechanical properties. Figure 5 illustrates the compression curves of the lattice samples with the same sample size and different cell sizes and relative densities. It can be seen that the compression curves of the lattice samples with a cell size of 7.5 mm are characterized by smooth contours and demonstrate high compressive strengths, indicating the advantages associated with smaller cell sizes. On the contrary, the lattice samples with a larger cell showed poorer mechanical properties because of a more severe stress concentration when subjected to the same external loads. In the elastic deformation stage, it is found that the trend of stress variation with strain for lattice samples with different cell sizes is essentially similar, which means that their structural efficiencies are approximately equal. However, the samples with larger cells exhibit earlier failure and damage, leading to a noticeable decline in the curve. Therefore, under the same sample size, the stress–strain curve of the sample with a smaller unit cell is smoother, the compressive strength is higher, and the strain at fracture is also greater.

**Figure 6:**
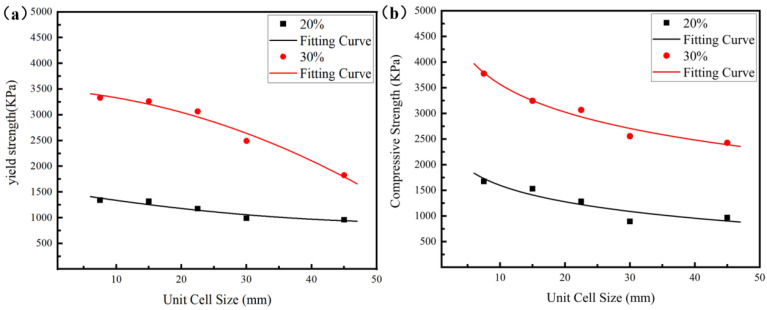
The yield strength (**a**) and compressive strength (**b**) of diamond lattices with relative densities of 20% and 30%.

To more intuitively compare the influence of the cell size and relative density on the mechanical properties of the lattice structure, Figure 6 illustrates the evolution trends of both yield strength and compressive strength as functions of the cell size and relative density. It can be found that the sample with a density of 30% exhibits a 142% increase in strength compared to the sample with a density of 20%, which is consistent with the Gibson–Ashby model, as follows;

(5) $$\sigma^{*}=C\sigma_{s}{\left(\frac{\rho^{*}}{\rho_{s}}\right)}^{n}$$

Here, $\sigma^{*}$ represents the lattice strength, $\sigma_{s}$ represents the strength of the material, $\rho^{*}$ represents the density of the lattice, $\rho_{s}$ represents the theoretical density of the material, $\rho^{*}/\rho_{s}$ is the relative density of the lattice, and *C* and *n* are constants related to the material and the lattice structure. There is an increase in lattice strength caused by the increase in relative density mainly due to two reasons. On the one hand, an increase in relative density can increase the elastic modulus, as shown in Figure 5, thereby enhancing the ability of the lattice structure to resist external forces under the same strain. On the other hand, increasing the relative density means that more material is used, thus increasing the overall structural strength according to the Gibson–Ashby model [39,40,41].

**Figure 7:**
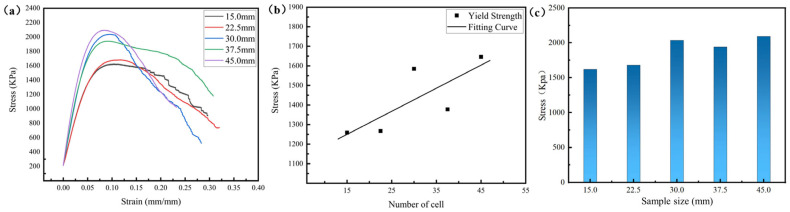
Mechanical properties of the lattice samples with different sample sizes. (**a**) Compression curves of diamond lattice; (**b**,**c**) strength variation rule.

Figure 7 illustrates the compression curves and mechanical properties of the lattice samples with the same cell size but different sample sizes. It can be seen that the lattice samples with a sample size of 15 mm exhibited the poorest compressive strength. The lattice sample with a size of 45 mm showed a 30.74% increase in strength compared to the sample with a size of 15 mm. A larger sample size means a greater number of cells, and the external load can be more evenly distributed over more cells, thereby alleviating the degree of stress concentration and increasing the strength of the sample to a certain extent.

It can be seen from Figure 5, Figure 6 and Figure 7 and the corresponding analysis that the lattice structures with greater relative density, smaller cell size, and larger sample size exhibit higher yield strength and compressive strength. The influence of a larger sample size on the mechanical properties of the lattice structure is similar to that of a smaller cell size. Both of them can enable the lattice structure to contain more cells. Having more cells in the lattice structure is like having more grains in crystalline materials, which can alleviate the stress concentration in the structure/material to a certain extent and improve the strength of the structure/material.

### 3.2. Stress Distribution of Diamond Lattice Structures

In Section 3.1, this work reveals the influence law of structural parameters on the mechanical properties of the lattice through experiments, but it still lacks quantitative data to clarify the influence mechanism of structural parameters. Here, we simulate the stress distribution of the lattices with different structural parameters and further calculate the structural efficiency and stress concentration coefficient of the lattice structure based on the simulation results. Given that the strength of the lattice is determined by the product of the structural efficiency, stress concentration coefficient, relative density, and material strength, and only a single material is used in this study, the influence of the material strength variation can be ignored. Thus, under the same relative density condition, by comparing the changing trends of the structural efficiency and stress concentration coefficient, the mechanism of the effect of structural parameters on the lattice strength can be revealed.

**Figure 8:**
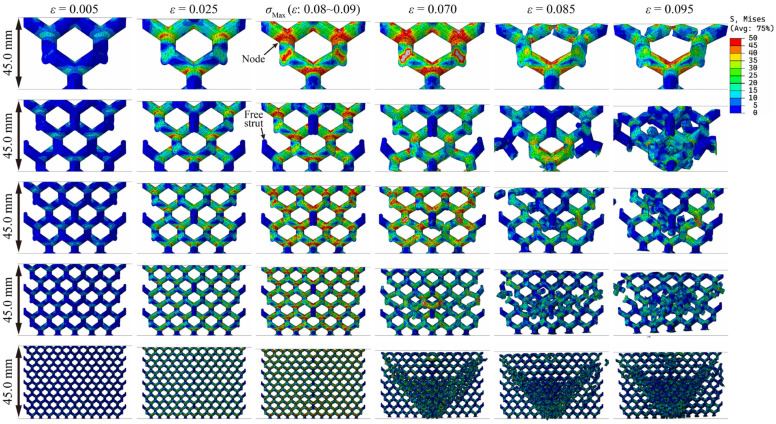
Stress distributions and fracture behavior of resin diamond lattice samples with the same sample sizes but different cell sizes (relative density 20%).

**Figure 9:**
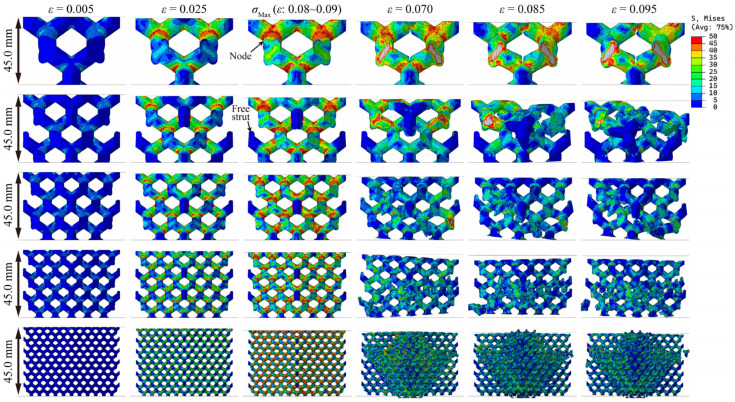
Stress distributions and fracture behavior of resin diamond lattice samples with the same sample sizes but different cell sizes (relative density 30%).

**Figure 10:**
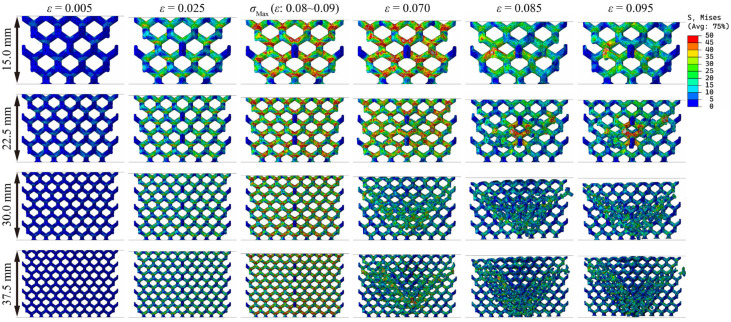
Stress distributions and fracture behavior of resin diamond lattice samples with the same cell sizes but different sample sizes.

Figure 8, Figure 9 and Figure 10 show the stress distribution of lattice structures with different structural parameters. The results show that when the lattice structure contains only one cell (sample size = cell size), all the struts of the lattice are subjected to external forces, and stress concentration occurs at the nodes, leading to fractures. When the lattice structure contains multiple cells, there are inevitably free struts at the lattice boundaries, which hardly bear external forces during the entire compression service process, and there is still obvious stress concentration at the nodes. Moreover, as the number of cells increases rapidly, the macroscopic cracks of the lattice gradually expand along the diagonal (known as the shear zone). The lattice near the shear zone is almost completely fractured, while the lattice far from the shear zone is basically undamaged.

By comparing Figure 8 and Figure 9, it can be observed that the stress concentration of the lattice structure primarily occurs in the node regions. It is worth noting that with the increase in relative density, the stress distribution does not change significantly. If the increase in lattice strength is explained only from the perspective of material usage, as shown in Formula (4), there should be a linear relationship between lattice strength and relative density, which is in obvious contradiction with the Gibson–Ashby model. Therefore, it can be inferred that the two key parameters, structural efficiency and the stress concentration coefficient, are very likely to be significantly affected by changes in relative density, which is difficult to discover through the visual comparison of Figure 8 and Figure 9. A larger structural efficiency means that the lattice can exhibit higher strength under the same stress level, while a higher stress concentration coefficient indicates that when the lattice bears the maximum load it can withstand, its stress level is higher. Greater structural efficiency and a higher stress concentration coefficient correspond to greater lattice strength.

### 3.3. Structural Efficiency and Stress Concentration Coefficient

**Figure 11:**
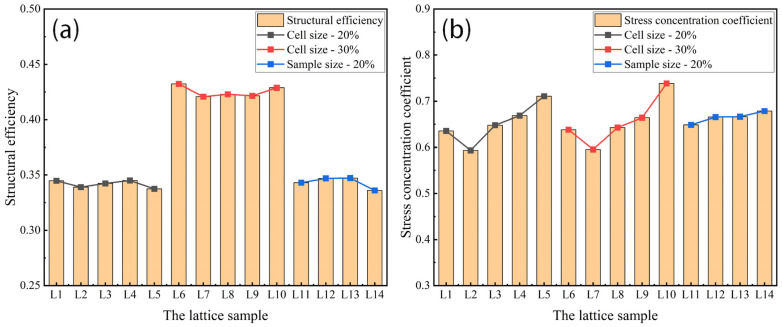
Structural efficiency and stress concentration coefficient of lattice samples with different relative densities, cell sizes, and sample sizes. (**a**) Structural efficiency; (**b**) stress concentration coefficient.

The difference in the stress distribution between Figure 8 and Figure 9 is already subtle, while the stress distribution characteristics between Figure 8 and Figure 10 are virtually indistinguishable through visual comparison, making it difficult to accurately assess the influence mechanism of cell size and sample size on mechanical properties solely by relying on image analysis methods. In contrast, the key parameters calculated based on Formula (4) (including relative density, structural efficiency, and the stress concentration coefficient) can provide a more reliable basis for quantitative analysis, as shown in Table 3 and Figure 11. The results indicate that the structural efficiency is closely related to the relative density, while the stress concentration coefficient is mainly affected by the cell size. This is because the changes in cell size and sample size did not alter the structural characteristics of the lattice. The orientation of the external force and the aspect ratio of the struts remained unchanged, resulting in the structural efficiency of these lattices being similar. When the sample size is 45 mm, the structural efficiency hardly increases with the cell size decreasing, while the stress concentration coefficient increases gradually, except when the cell size is 30 mm due to incomplete lattice structures.

When the relative density and cell size are constant (e.g., samples L11–L14), as the sample size increases, the structural efficiency remains relatively stable (0.3361~0.3473), which is highly consistent with the structural efficiency of samples L1–L5 (0.3375~0.3451). It can be inferred that the structural efficiency is mainly influenced by two key parameters, namely the cell type and the relative density, and does not vary with changes in the sample size or the cell size. However, the stress concentration coefficient increases gradually as the sample size increases due to a higher number of cells, resulting in improved mechanical properties of lattice structures with a larger sample size, as shown in Figure 7.

It is worth noting that when the cell size is equal to the sample size, there are no free struts in the lattice, and its stress concentration coefficient is improved. In addition, the designed relative density (~18.85% or 27.82%) is the smallest due to the removal of free struts. The decrease in relative density and the increase in the stress concentration coefficient result in similar mechanical properties for samples L1 and L2, as illustrated in Figure 7. Similarly, samples L6 and L7 exhibit comparable mechanical properties.

Lattice samples with a greater relative density, smaller cell size, and larger sample size exhibited higher mechanical properties. This trend can be attributed to their having more material, higher structural efficiency, and improved uniformity of the stress distribution [42,43], which results in an increase in compressive properties according to Formula (4).

## 4. Conclusions

The objective of this study is to explore the impact of the relative density, cell size, and sample size on the mechanical properties of diamond lattice structures. The structural efficiency and stress concentration coefficient of diamond structures with different relative densities, cell sizes, and sample sizes were evaluated to reveal the mechanism by which structural parameters affect lattice strength. The relevant conclusions could guide the analysis of lattice stress distribution and the design of lattice structure parameters.

1. The findings of this study emphasize the obvious influence of relative density, cell size, and sample size on the mechanical properties of diamond lattice structures.
2. The lattice samples with a relative density of 30% exhibited a 142% increase in compressive strength compared to those with a relative density of 20%.
3. Greater relative density results in higher structural efficiency, and both greater relative density and higher structural efficiency lead to an increase in the compressive strength of diamond lattice structures with a greater relative density.
4. Both smaller cell sizes and larger sample sizes can enable a lattice structure to contain more cells, thereby increasing the stress concentration coefficient and improving the mechanical properties of the lattices to a certain extent.
5. The structural efficiency is closely related to the cell type and relative density, which hardly change as the cell size or sample size changes.

## Figures and Tables

**Table 1:** The structural parameters of diamond lattice samples.

The Lattices with Different Cell Sizes and Relative Densities			The Lattices with Different Sample Sizes		
Sample Size	Cell Size	Relative Density	Sample Size	Cell Size	Relative Density
45 mm	45 mm	20% and 30%	45 mm	7.5 mm	20%
45 mm	30 mm	20% and 30%	37.5 mm	7.5 mm	20%
45 mm	22.5 mm	20% and 30%	30 mm	7.5 mm	20%
45 mm	15 mm	20% and 30%	22.5 mm	7.5 mm	20%
45 mm	7.5 mm	20% and 30%	15 mm	7.5 mm	20%

**Table 2:** Physical properties of the photosensitive resin.

Fracture Strain	Displacement at Failure	Density	Young’s Modulus	Poisson’s Ratio	Yield Stress 1	Plastic Strain 1	Yield Stress 2	Plastic Strain 2
0.16	0.0025	1.3 × 10^−9^ t/mm^3^	2250 MPa	0.3	39.3 MPa	0	45.5	0.16

**Table 3:** The structural parameters of diamond lattice samples and the corresponding results.

Sample	Sample Sizes (mm)	Cell Size (mm)	Relative Density (%)	Structural Efficiency	Stress Concentration Coefficient	Simulated Strength (KPa)	Calculated Strength by Formula (4) (KPa)
L1	45.0	45.0	18.85	0.3449	0.6356	1798	1775
L2	45.0	30.0	19.98	0.3391	0.5934	1545	1726
L3	45.0	22.5	19.35	0.3425	0.6482	1746	1844
L4	45.0	15.0	19.41	0.3451	0.6688	1796	1924
L5	45.0	7.5	19.41	0.3375	0.7110	1894	2000
L6	45.0	45.0	27.82	0.4323	0.6383	3418	3296
L7	45.0	30.0	29.72	0.4208	0.5954	3362	3197
L8	45.0	22.5	28.96	0.4230	0.6429	3578	3382
L9	45.0	15.0	29.16	0.4216	0.6642	3738	3507
L10	45.0	7.5	28.50	0.4280	0.7384	4182	3867
L11	15.0	7.5	19.33	0.3431	0.6488	1819	1847
L12	22.5	7.5	19.39	0.3470	0.6657	1872	1924
L13	30.0	7.5	19.41	0.3473	0.6663	1901	1928
L14	37.5	7.5	19.42	0.3361	0.6785	1953	1902

## Data Availability

The Python script and the Abaqus inp file can be found on the site https://github.com/Zuleiliang/Mises-Volume-curve-for-lattice.git (accessed on 1 June 2025). Other data will be made available on request.
